# A *pri-miR-218* variant and risk of cervical carcinoma in Chinese women

**DOI:** 10.1186/1471-2407-13-19

**Published:** 2013-01-15

**Authors:** Ting-Yan Shi, Xiao-Jun Chen, Mei-Ling Zhu, Meng-Yun Wang, Jing He, Ke-Da Yu, Zhi-Ming Shao, Meng-Hong Sun, Xiao-Yan Zhou, Xi Cheng, Xiaohua Wu, Qingyi Wei

**Affiliations:** 1 Cancer Institute, Fudan University Shanghai Cancer Center, Shanghai, China; 2 Department of Gynecologic Oncology, Fudan University Shanghai Cancer Center, Shanghai, China; 3 Department of Breast Surgery, Fudan University Shanghai Cancer Center, Shanghai, China; 4 Department of Pathology, Fudan University Shanghai Cancer Center, Shanghai, China; 5 Department of Oncology, Shanghai Medical College, Fudan University, Shanghai, China; 6 Department of Epidemiology, The University of Texas M.D, Anderson Cancer Center, Houston, Texas, USA

**Keywords:** Case–control study, Cervical cancer, *LAMB3-miR-218* pathway, Polymorphism, Genetic susceptibility

## Abstract

**Background:**

MicroRNA (miRNA)-related single nucleotide polymorphisms (SNPs) may compromise miRNA binding affinity and modify mRNA expression levels of the target genes, thus leading to cancer susceptibility. However, few studies have investigated roles of miRNA-related SNPs in the etiology of cervical carcinoma.

**Methods:**

In this case–control study of 1,584 cervical cancer cases and 1,394 cancer-free female controls, we investigated associations between two *miR-218*-related SNPs involved in the *LAMB3-miR-218* pathway and the risk of cervical carcinoma in Eastern Chinese women.

**Results:**

We found that the *pri-miR-218* rs11134527 variant GG genotype was significantly associated with a decreased risk of cervical carcinoma compared with AA/AG genotypes (adjusted OR=0.77, 95% CI=0.63-0.95, *P*=0.015). However, this association was not observed for the *miR-218* binding site SNP (rs2566) on *LAMB3*. Using the multifactor dimensionality reduction analysis, we observed some evidence of interactions of these two SNPs with other risk factors, especially age at primiparity and menopausal status, in the risk of cervical carcinoma.

**Conclusions:**

The *pri-miR-218* rs11134527 SNP was significantly associated with the risk of cervical carcinoma in Eastern Chinese women. Larger, independent studies are warranted to validate our findings.

## Background

MicroRNAs (miRNAs) are single-stranded 21–23 nucleotide (nt) long endogenous noncoding RNAs that regulate the mRNA expression of numerous target genes [1]. Disregulation of these target genes could alter biological processes as a result of either degradation of target mRNAs or repression of their translation by miRNA binding to their 3^′^-untranslated regions (UTRs) [2]. Accumulated data have shown that the deregulation of miRNAs is involved in cell differentiation, proliferation, apoptosis and carcinogenesis [3]. MiRNAs include primary (pri-), precursor (pre-) and mature miRNA, in which single nucleotide polymorphisms (SNPs) of these miRNAs or in their binding sites on their target genes may compromise miRNA binding affinity and change mRNA expression levels of the target genes, thus leading to cancer susceptibility [4,5]. Several recent studies have indicated that miRNA-related SNPs, especially those located at miRNA binding sites or miRNAs themselves, can remarkably alter the biogenesis and/or function of the corresponding miRNAs and thus the risk of human cancers [4,6].

Cervical carcinoma is the third most commonly diagnosed cancer and the fourth leading cause of cancer deaths in women worldwide, accounting for 9% (529,800) of the new cancer cases and 8% (275,100) of the cancer deaths among women in 2008 [7]. More than 85% of these cases and deaths occur in developing countries, including China [7]. Invasive cervical cancer can be divided into two major histological types of squamous cell carcinoma (SCC) and adenocarcinoma, and SCC accounts for about 85% of the cases [8,9]. A large body of research in molecular epidemiology supports the hypothesis that persistent infection with oncogenic human papillomavirus (HPV), especially high-risk HPV types, is the primary cause of cervical carcinoma, deemed as a necessary cause for the disease [7,10].

Recent studies have found that the expression levels of *miR-218* were associated with infection of high-risk HPV involved in the pathogenesis of cervical cancer [11]. Specifically, in high-risk HPV16-positive cell lines, the upregulation of E6 oncoprotein could reduce the *miR-218* expression; in contrast, the RNA interference of E6 oncogene increased the *miR-218* expression [12]. Moreover, the *Laminin 5 β3* (*LAMB3*) gene has been found to be one of transcriptional targets of *miR-218*[12]. *LAMB3* was expressed in many epithelial tissues and was involved in tumor microenvironment by increasing carcinoma cell migration [13]. Others reported that *LAMB3* might upregulate the expression levels of the HPV16 E6 oncoprotein though *miR-218*[12]. Therefore, the *LAMB3-miR-218* pathway may be involved in the process of high-risk HPV infection and thus contribute to cervical carcinogenesis. However, its intrinsic mechanisms are still unclear. It is likely that miRNAs and related genetic variations may have effects on cancer development [6]. To date, only two reported studies have investigated the associations between three miRNA-related SNPs and the risk of cervical carcinoma [6,14], two of which (i.e., *pri-miR-218* rs11134527 and *LAMB3* rs2566) are found to be associated with altered risk of cervical cancer in a Chinese Han population [6]. To further test the hypothesis that miRNA-related SNPs involved in the *LAMB3-miR-218* pathway contribute to cervical cancer risk, we performed a case–control study with a much larger sample size to validate the reported associations with cervical cancer risk in Eastern Chinese women.

## Methods

### Study subjects

The study population consisted of 1,584 cervical carcinoma patients, who had been operated between February 2008 and March 2011 in Fudan University Shanghai Cancer Center (FUSCC). The tumors were histopathologically confirmed independently as primary cervical carcinoma by two gynecologic pathologists as routine diagnosis at FUSCC. An additional 1,394 cancer-free female controls were enrolled from women who had come to the Outpatient Department of Breast Surgery at FUSCC for breast cancer screening and agreed to participate in this study. These female controls, with the selection criteria including no individual history of cancer, were genetically unrelated and frequency matched to the cases on age (± 5 years) and residential areas in Eastern China.

During an in-person survey, all potential subjects were interviewed to identify their willingness to participate in this study. As a result, a response rate for the cases and controls was of approximate 95% and 95%, respectively. Because the vast majority of Chinese women are non-smokers and non-drinkers, our study populations were restricted to women who did not smoke cigarettes or drink alcohol. For the cases, detailed clinico-pathologic information was extracted from the patients′ electronic database of FUSCC, including tumor histology [15], FIGO stage (International Federation of Gynecology and Obstetrics, 2009), tumor size (i.e., the size of the primary tumor was the largest tumor diameter), pelvic lymph node (LN) metastasis, lympho-vascular space invasion (LVSI), depth of cervical stromal invasion and the expression of estrogen receptor (ER) and progesterone receptor (PR). Each participant provided a one-time 10 ml of venous blood sample (after the diagnosis and before the initiation of treatment for the cases), and samples were kept frozen till DNA extraction for genotyping. All samples were obtained from tissue bank of FUSCC. The research was approved by the Institutional Review Board of FUSCC, and a written informed consent was obtained from all recruited individuals. Each clinical investigation was conducted according to the principles expressed in the Declaration of Helsinki consent.

### SNP selection and genotyping

The SNPs were selected from the NCBI dbSNP database (http://www.ncbi.nlm.nih.gov/projects/SNP) and the International HapMap Project database (http://hapmap.ncbi.nlm.nih.gov/) based on four criteria: 1) located at the *pri-miR-218* gene region or 3^′^-UTR of the *LAMB3* gene, 2) minor allele frequency (MAF) ≥ 5% in Chinese Han populations, 3) with low linkage disequilibrium by using an *r*^2^ threshold of < 0.8 for each other, and 4) predicted as potentially functional SNPs by SNP function prediction (FuncPred) software from National Institute of Environmental Health Sciences (http://snpinfo.niehs.nih.gov/snpfunc.htm). As a result, only two reported SNPs (i.e., *pri-miR-218* rs11134527 and *LAMB3* rs2566) were selected, because *pri-miR-218* rs11134527 was predicted to be functional and *LAMB3* rs2566 was the only one SNP residing in the *miR-218* binding site. Genomic DNA was obtained from the whole blood, and the Taqman assay was performed for genotyping, as described previously [16,17]. Four negative controls (without DNA template), duplicated positive controls and eight repeat samples were included in each 384-fomate for the quality control. As a result, the mean genotyping rate was 99.3%, and the discrepancy rate in all positive controls (i.e., duplicated samples, overlapping samples from previous studies and samples randomly selected to be sequenced) was less than 0.1%.

### Multifactor dimensionality reduction (MDR) analysis

To further explore high-order gene-environment interactions that were individually involved in cervical cancer risk, we performed the MDR analysis, as described previously [17,18]. This approach was used to find the main factor and the combination of multiple factors (in this case, SNPs and environmental risk factors) that were significantly associated with cancer risk. As a result, the model that minimized the prediction error and maximized the cross-validation consistency (CVC) was chosen. To reduce the probability of bias, we used different random seeds to repeat the complete analysis for 10 times, and permutated the status of cases and controls in the data set then repeated the test 1000 times under the null hypothesis of no association. This analysis was performed by using the MDR V2.0 beta 8.2 program (http://www.multifactordimensionalityreduction.org/).

### Statistical analysis

The differences in selected variables between cervical carcinoma cases and female controls were evaluated by the Pearson's χ^2^-test. The associations of genotypes with the risk of cervical carcinoma were estimated by computing odds ratios (ORs) and their 95% confidence intervals (CIs) from both univariate and multivariate logistic regression models, with or without adjustment for age, age at primiparity, menopausal status and body mass index (BMI) [19]. The associations of SNP genotypes with cervical carcinoma risk were also stratified by demographic and clinico-pathologic variables. We also performed homogeneity test and logistic regression analysis to estimate and compare the risks between the strata and interactions between two factors, respectively. For all significant genetic effects observed in our study, we calculated the false-positive report probability (FPRP) with prior probabilities of 0.0001, 0.001, 0.01, 0.1 and 0.25 to test for false-positive associations [20]. A FPRP value < 0.2 was considered a noteworthy and indicated a remained robust association for a given prior probability. Statistical power was estimated to detect an OR of 1.50/0.67 (for a risk/protective effect), with an α level equal to the observed *P* value [20]. All statistical analyses were performed with SAS software (version 9.1; SAS Institute, Cary, NC), unless stated otherwise. All *P* values were two-sided with a significance level of *P* < 0.05.

## Results

Among all studied subjects, 19 cases and three controls failed to be genotyped after repeated assays. Thus, the final analysis included 1,565 cases and 1,391 controls. As showed in Additional file 1: Table S1, there were no significant differences in the distributions of age between the cases and the controls with similar mean ages of 45.8 (± 9.8) and 46.1 (± 8.9) years, respectively (*P*=0.226). The cases were more likely to be premenopausal (72.5% *vs.* 60.5%), thinner (BMI < 25 kg/m^2^, 78.2% *vs.* 65.9%) and younger at primiparity (≤ 24 yr, 63.2% *vs.* 51.0%) than the controls. Because the differences in age at primiparity, menopausal status and BMI were significant between cases and controls (all *P*<0.001), these variables were further adjusted for any residual confounding effect in later multivariate logistic regression analyses.

The genotype frequencies of the *pri-miR-218* rs11134527 and *LAMB3* rs2566 SNPs as well as their associations with the risk of cervical carcinoma are summarized in Table 1. All observed genotype distributions in the 1,391 controls agreed with the Hardy-Weinberg equilibrium (HWE, *P*=0.083 and 0.094 for rs11134527 and rs2566, respectively). In the recessive genetic model, the *pri-miR-218* rs11134527 variant GG genotype was significantly associated with a decreased risk of cervical carcinoma compared with the AA and AA/AG genotypes (adjusted OR=0.79 and 0.77, 95% CI=0.63-0.99 and 0.63-0.95, *P*=0.039 and 0.015, respectively). However, this association was not observed for the *LAMB3* rs2566 SNP.

**Table 1:** **Logistic regression analysis of associations between genotypes of the *LAMB3-miR-218 *pathway and cervical cancer risk**

**Variants Genotypes**	**Cases**	**Controls**	P*	**Crude OR**	***P***	**Adjusted OR**	P**
	**(N=1565)**	**(N=1391)**		**(95% CI)**		**(95%CI)**	
***pri-miR-218 *rs11134527**							
AA	588 (37.6)	512 (36.8)	0.085	1.00		1.00	
AG	752 (48.1)	638 (45.9)		1.03 (0.88-1.20)	0.748	1.03 (0.87-1.22)	0.705
GG	225 (14.4)	241 (17.3)		0.81 (0.65-1.01)	0.061	0.79 (0.63-0.99)	**0.039**
AG/GG	977 (62.4)	879 (63.2)	0.668^a^	0.97 (0.83-1.12)	0.668	0.96 (0.82-1.13)	0.648
Additive model				0.93 (0.84-1.03)	0.148	0.92 (0.82-1.02)	0.111
Recessive model			**0.028**^b^	0.80 (0.66-0.98)	**0.028**	0.77 (0.63-0.95)	**0.015**
***LAMB3 *rs2566**							
CC	667 (42.6)	570 (41.0)	0.431	1.00		1.00	
CT	709 (45.3)	663 (47.7)		0.91 (0.78-1.07)	0.252	0.89 (0.76-1.05)	0.165
TT	189 (12.1)	158 (11.4)		1.02 (0.81-1.30)	0.857	0.94 (0.73-1.21)	0.642
CT/TT	898 (57.4)	821 (59.0)	0.366^a^	0.94 (0.81-1.08)	0.367	0.90 (0.77-1.05)	0.186
Additive model				0.98 (0.88-1.09)	0.707	0.94 (0.84-1.06)	0.325
Recessive model			0.545^b^	1.07 (0.86-1.34)	0.546	1.00 (0.79-1.27)	1.000

OR, odds ratio; CI, confidence interval.

* *χ*^*2*^ test for genotype distributions between cases and controls;

** Adjusted for age, age at primiparity, menopausal status, BMI in logistic regression models;

^a^ for dominant genetic models;

^b^ for recessive genetic models.

The results were in **bold**, if *P* < 0.05.

In stratification analyses, as showed in Table 2, under a recessive genetic model, a decreased cervical carcinoma risk associated with the *pri-miR-218* rs11134527 GG genotype was more evident in women who were younger at primiparity (≤ 24 yr, adjusted OR=0.73, 95% CI=0.56-0.96, *P*=0.022) or premenopausal (adjusted OR=0.73, 95% CI=0.57-0.94, *P*=0.013), which was also observed for subgroups of SCC, FIGO stage I, stage II, positive pelvic LN, positive LVSI, deep cervical stromal invasion (> 1/2) and negative expression of ER and PR (*P*=0.008, 0.008, 0.028, 0.002, 0.008, 0.022, 0.011 and 0.014, respectively). However, homogeneity tests suggested that there was no difference in risk estimates between the strata (Table 2), and no statistical evidence for interactions between the genotypes and these variables on the risk of cervical carcinoma (Additional file 1: Table S2).

**Table 2:** **Stratification analysis for associations between genotypes of the *LAMB3-miR-218 *pathway and cervical cancer risk in the recessive genetic model**

**Variables**	**rs11134527**		Adjusted OR* (95% CI)	P*	P**	**rs2566**		Adjusted OR* (95% CI)	P*	P**
	**(cases/controls)**					**(cases/controls)**				
	**AA/AG**	**GG**				**CC/CT**	**TT**			
**Age, years**										
≤46 (Mean)	747/623	136/131	0.84 (0.64-1.11)	0.215	0.364	774/669	109/85	1.02 (0.74-1.40)	0.919	0.740
>46 (Mean)	593/527	89/110	0.77 (0.56-1.06)	0.111		602/564	80/73	1.00 (0.69-1.45)	0.995	
**Age at primiparity, years**										
≤24 (Mean)	797/568	136/131	0.73 (0.56-0.96)	**0.022**	0.452	822/625	111/74	1.12 (0.82-1.54)	0.482	0.460
>24 (Mean)	468/566	76/106	0.86 (0.62-1.20)	0.386		481/591	63/81	0.91 (0.63-1.32)	0.621	
**Menopausal status**										
Premenopausal	962/685	164/155	0.73 (0.57-0.94)	**0.013**	0.425	986/743	140/97	1.00 (0.75-1.34)	0.981	0.635
Postmenopausal	366/463	61/86	0.90 (0.62-1.32)	0.600		381/488	46/61	1.09 (0.70-1.70)	0.696	
**BMI, kg/m**^**2**^										
< 25	1026/759	175/157	0.79 (0.62-1.00)	0.054	0.715	1061/810	140/106	1.00 (0.75-1.32)	0.973	0.739
≥ 25	288/390	47/84	0.74 (0.50-1.12)	0.152		295/422	40/52	1.02 (0.64-1.63)	0.939	
**Histology**										
CINIII	129/1150	32/241	1.06 (0.68-1.66)	0.789	0.169	137/1233	24/158	1.32 (0.80-2.16)	0.274	0.409
SCC	1068/1150	170/241	0.74 (0.59-0.92)	**0.008**		1096/1233	142/158	0.95 (0.73-1.22)	0.673	
Non-squamous	138/1150	23/241	0.75 (0.46-1.22)	0.240		138/1233	23/158	1.18 (0.71-1.96)	0.526	
**FIGO stage**										
I	633/1150	97/241	0.70 (0.53-0.91)	**0.008**	0.796	645/1233	85/158	0.94 (0.70-1.27)	0.689	0.341
II	464/1150	75/241	0.72 (0.54-0.97)	**0.028**		478/1233	61/158	0.96 (0.69-1.35)	0.830	
III~IV	43/1150	5/241	0.43 (0.15-1.28)	0.129		39/1233	9/158	1.97 (0.83-4.71)	0.126	
**Tumor size, cm**										
< 4	801/1150	139/241	0.78 (0.61-0.99)	**0.043**	0.695	840/1233	100/158	0.88 (0.66-1.16)	0.365	0.100
≥ 4	428/1150	69/241	0.71 (0.53-0.97)	**0.031**		426/1233	71/158	1.25 (0.91-1.72)	0.176	
**Pelvic LN**										
Negative	965/1150	174/241	0.83 (0.66-1.04)	0.098	0.095	1002/1233	137/158	1.00 (0.77-1.29)	0.970	0.819
Positive	309/1150	39/241	0.55 (0.37-0.80)	**0.002**		308/1233	40/158	0.96 (0.64-1.42)	0.828	
**LVSI**										
Negative	750/1150	132/241	0.80 (0.63-1.02)	0.073	0.305	783/1233	99/158	0.93 (0.70-1.24)	0.632	0.379
Positive	390/1150	56/241	0.64 (0.46-0.89)	**0.008**		387/1233	59/158	1.06 (0.75-1.50)	0.755	
**Depth of cervical stromal invasion**										
≤ 1/2	584/1150	99/241	0.75 (0.57-0.98)	0.037	0.898	598/1233	85/158	1.08 (0.81-1.45)	0.602	0.709
> 1/2	670/1150	111/241	0.74 (0.57-0.96)	**0.022**		690/1233	91/158	0.94 (0.70-1.26)	0.660	
**ER expression**										
Negative	647/1150	102/241	0.71 (0.55-0.92)	**0.011**	0.146	671/1233	78/158	0.85 (0.62-1.15)	0.289	0.365
Positive	50/1150	13/241	1.01 (0.51-1.98)	0.982		54/1233	9/158	1.35 (0.65-2.81)	0.428	
**PR expression**										
Negative	677/1150	110/241	0.73 (0.56-0.94)	**0.014**	0.407	703/1233	84/158	0.87 (0.65-1.18)	0.370	0.836
Positive	20/1150	5/241	0.94 (0.32-2.80)	0.911		22/1233	3/158	1.13 (0.33-3.84)	0.851	

OR, odds ratio; CI, confidence interval; BMI, body mass index; FIGO, International Federation of Gynecology and Obstetrics; CIN, cervical intraepithelial neoplasia; SCC, squamous cell carcinoma; LN, lymph node; LVSI, lympho-vascular space invasion; ER, estrogen receptor; PR, progesterone receptor.

* Logistic regression models with adjustment for age, age at primiparity, menopausal status and BMI;

** Homogeneity test.

The results were in **bold**, if *P* < 0.05.

We calculated the FPRP values for all the observed significant associations. When the assumption of prior probability was 0.1, the association with the *pri-miR-218* rs11134527 (GG *vs.* AA/AG) was still noteworthy in subgroups of premenopausal, SCC, FIGO stage I and positive pelvic LN (FPRP=0.189, 0.111, 0.163 and 0.153, respectively) (Additional file 1: Table S3).

**Figure 1:**
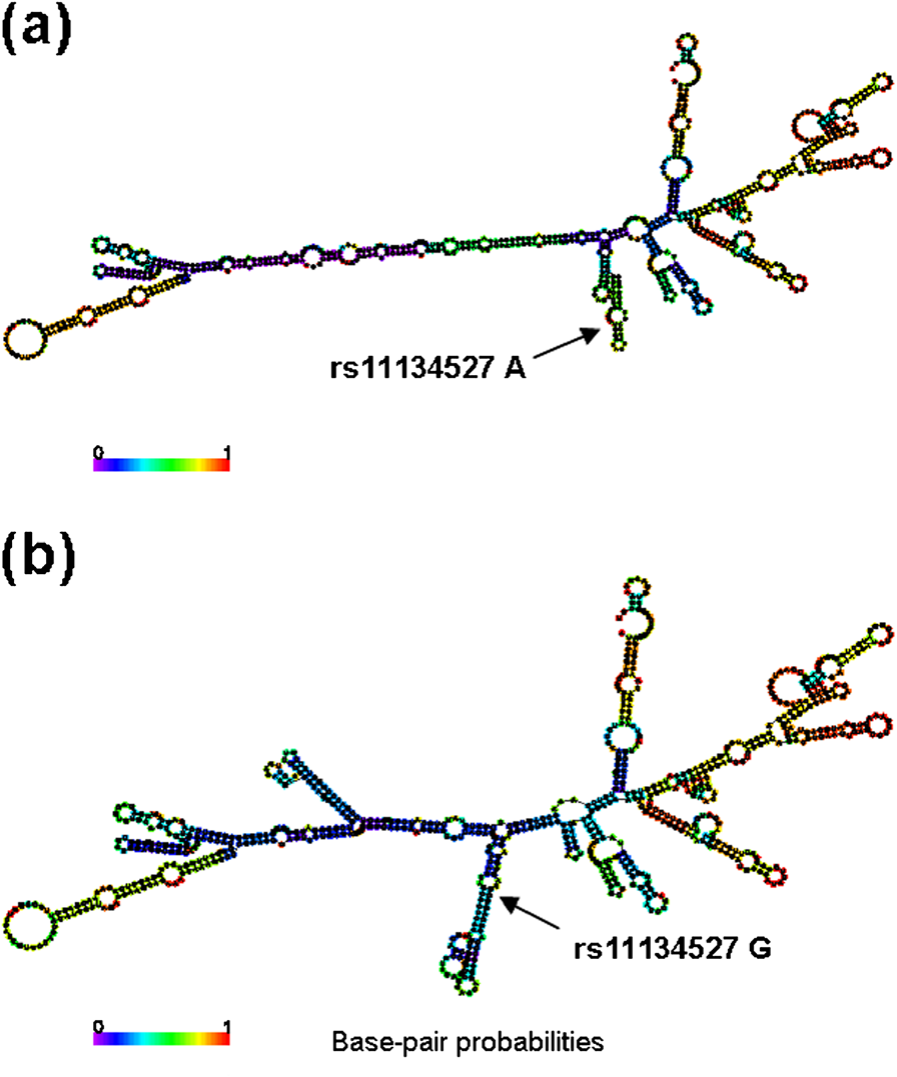
**The secondary structures of the *pri-miR-218 *mRNA.** These structures were predicted by inputting two 801-nt long *pri-miR-218* DNA sequences centering the rs11134527 locus into RNAfold, with either (**a**) the rs11134527-A or (**b**) rs11134527-G allele. The figures and the values of minimum free energy were generated by RNAfold (http://rna.tbi.univie.ac.at).

To further explore whether the *pri-miR-218* rs11134527 variant could alter the local second structure of the *pri-miR-218* mRNA, we performed the RNAfold online tool that is an online RNA secondary structure prediction software based on the minimum free energy (MFE) and found that the MFE changed from −182.5 kcal/mol to −126.0 kcal/mol when the nucleotide at the *pri-miR-218* rs11134527 locus changed from A to G (Figure 1).

Moreover, using the MDR analysis and including these two SNPs and three risk factors, we found that age at primiparity was the best one-factor model with the highest CVC (100%) and the lowest prediction error (43.2%) among all five discrete factors. Intriguingly, the five-factor model had a maximum CVC (100%) and a minimum prediction error (38.6%), which showed a better prediction than one factor (Table 3).

**Table 3:** **MDR analysis for the cervical cancer risk prediction with and without *LAMB3-miR-218 *pathway genotypes**

**Number of risk factors**	**Best interaction models by MDR analysis**	**Cross- validation**	**Average prediction error**	***P *for permutation test**
1	age at primiparity	100/100	43.2%	<.0001
2	age at primiparity, BMI	100/100	40.4%	<.0001
3	age at primiparity, menopausal status, BMI	100/100	39.2%	<.0001
4	age at primiparity, menopausal status, BMI, rs2566	83/100	39.2%	<.0001
**5**	**age at primiparity, menopausal status, BMI, rs11134527, rs2566**	**100/100**	**38.6%**	**<.0001**

MDR, multifactor dimensionality reduction.

The multi-locus model with maximum cross-validation consistency and minimum prediction error rate is indicated in **bold.**

## Discussion

In this relatively large hospital-based case–control study of 1,584 cervical cancer cases and 1,394 cancer-free female controls, we validated two previously reported significant miRNA-related SNPs involved in the *LAMB3-miR-218* pathway for the risk of cervical carcinoma in Chinese populations [6]. We found that the *pri-miR-218* rs11134527 variant GG genotype was significantly associated with a decreased risk of cervical carcinoma compared with the AA and AA/AG genotypes, and our sample size had a statistical power of 94.9% to detect such an association. Further RNAfold prediction analysis showed a MFE changed from −182.5 kcal/mol to −126.0 kcal/mol, when the nucleotide at the *pri-miR-218* rs11134527 locus changed from A to G, indicating that this variant may act as a functional SNP, which affects the miRNA binding process and contributes to cervical cancer susceptibility. However, for the other SNP (i.e., *LAMB3* rs2566), our data did not have statistical evidence to support its association with cervical cancer risk. Our sample size had 100% statistical power to detect an OR of 1.57 that was reported by Zhou et al. [6]. The inconsistency for the *LAMB3* rs2566 SNP between Zhou’s study and ours may be caused by differences in selection of subjects, different catchments of the hospitals and residential regions as well as different sample sizes.

Recent studies have demonstrated that miRNAs may function as tumor suppressors and/or oncogenes in human cancers [21,22], because elevated or decreased expression of miRNAs has been found in various tumor types, which may alter the regulation of mRNA expression. It is of note that miRNAs regulate gene expression by the sequence-specific binding to the target mRNA, and these binding processes may be affected by SNPs located in the miRNA complementary site [23]. Therefore, it is important to understand the functional and evolutionary significance of related genetic variations in determining expression of miRNAs and mRNAs that interact with each other as well as with environmental risk factors in the related biological processes [23,24].

It is well known that genetic variants may modify cancer risk associated with environmental factors. Although there were no two-factor interactions between genotypes and environmental factors, using the MDR analysis [18], we further explored high-order multiple-factor interactions in associations with cervical cancer risk and found that age at primiparity was the strongest risk predictor among all the risk factors considered. Meanwhile, the interaction between the variant genotypes and other risk factors appeared to modify the risk of cervical carcinoma, with the five-factor model being the best model.

*MiR-218,* is encoded by an intron of the *SLIT2* tumor suppressor gene [25], is known to be associated with the development and progression of several cancers [21,22]. The decreased level of the *miR-218* expression has been observed in cancers of the breast, ovary, lung and stomach [22,26,27], and its low expression level was also correlated with tumor stage, LN metastasis and poor prognosis in gastric cancer [27]. Recently, Martinez et al. reported a decreased expression level of *miR-218* (> 2 fold) in HPV-16 or 18 positive cervical cancer cell lines (i.e., SiHa, CaSki and HeLa) as well as in cervical tumor tissues [12]. They also demonstrated *miR-218* as a specific cellular target of high-risk HPV types [12], suggesting that the down-regulation of *miR-218* is likely linked to the process of HPV-associated tumorgenesis. Based on the Microcosm Targets tool software (http://www.ebi.ac.uk/enright-srv/microcosm/), the mature *miR-218* was found to have an effect on the mRNA expression regulation through more than 900 target genes, including *LAMB3*[12], *RICTOR*[28], *ROBO1*[27] and *BIRC5*[29], that may play important roles in cervical carcinogenesis. These genes were reported to participate in a number of cancer signaling pathways, such as the Wnt/β-catenin, ERK/MAPK and Notch pathways [30]. Laminin-5 has been found as a sensitive marker of early invasion of cervical lesions [31]. *LAMB3* that expressed in many epithelial tissues could induce carcinogenesis by increasing carcinoma cell migration and disturbing tumor microenvironment [13]. Moreover, *LAMB3* increased expression levels of the HPV16 E6 oncoprotein in cervical cancer cells and this process might be mediated by *miR-218*[12], which indicates a possible mechanism of the *LAMB3-miR-218* pathway involved in the development of cervical carcinoma.

It is known that the mRNA secondary structure is critical for mRNA-miRNA interactions and gene functions [32]. To investigate whether the *pri-miR-218* rs11134527 SNP could alter the local second structure of the *pri-miR-218* mRNA, we performed the RNAfold prediction analysis and found an obviously changed mRNA structure from rs11134527 allele A to G. These findings further suggest that germline genetic variations of *pri-miR-218*, such as rs11134527, may lead to an alteration of *miR-218* expression and affect the miRNA binding process and thus are associated with cervical cancer susceptibility.

Several limitations of our study need to be addressed. Firstly, this hospital-based case–control study may have selection bias and information bias, which may be minimized by frequency-matching cases and controls as well as the adjustment for potential confounding factors in the final analyses. Secondly, only two *miR-218*-related SNPs involved in the *LAMB3-miR-218* pathway (i.e., one in *pri-miR-218* and the other in *miR-218* binding site) were investigated in this study. Cancer is a complex and multifactorial disease, and any single SNP may not be sufficient for the prediction of the overall risk [33]. Future studies should include more genes and more SNPs, especially functional ones, associated with cervical cancer risk. Finally, we did not have enough information on other risk factors, especially HPV infection. This was because the hospital did not perform HPV and related subtype detection for the diagnosis of all cervical cancer cases, let alone for the female controls. A recent meta-analysis found that high-risk HPV16, 18 and 45 types accounted for a greater or equal proportion of HPV infections in cervical cancer, but not other high-risk HPV types, such as HPV33, 51 and 58 [34]. Therefore, HPV types could be confounders in estimating the risk associated with genetic factors.

## Conclusions

In summary, in the current case–control study of 1,584 cases and 1,394 controls, we found that the *pri-miR-218* rs11134527 SNP was associated with the risk of cervical carcinoma in Eastern Chinese women. Our findings suggest some possible interactions between genetic variations involved in the *LAMB3-miR-218* pathway and other risk factors for cervical carcinoma. However, well-designed prospective studies with larger sample sizes are required to validate our findings.

## Abbreviations

miRNA: microRNA;nt: Nucleotide;UTR: Untranslated region;pri-miRNA: Primary miRNA;pre-miRNA: Precursor miRNA;SNP: Single nucleotide polymorphism;SCC: Squamous cell carcinoma;HPV: Human papillomavirus;LAMB3: Laminin 5 β3;FUSCC: Fudan University Shanghai Cancer Center;FIGO: International Federation of Gynecology and Obstetrics;LN: Lymph node;LVSI: Lympho-vascular space invasion;ER: Estrogen receptor;PR: Progesterone receptor;MAF: Minor allele frequency;MDR: Multifactor dimensionality reduction;CVC: Cross-validation consistency;OR: Odds ratio;CI: Confidence interval;BMI: Body mass index;FPRP: False-positive report probability;HWE: Hardy-Weinberg equilibrium;MFE: Minimum free energy

## Competing interests

The authors declare that they have no competing interests.

## Authors’ contributions

Conception and design: QW, XW. In-person survey and data collection: XJC, XC, KDY, ZMS, MHS. Genotyping and Provision of study materials: TYS, MLZ, MYW, JH, XYZ. Data analysis and interpretation: TYS, QW. Manuscript writing: TYS, QW, XW. Final approval of manuscript: TYS, XJC, MLZ, MYW, JH, KDY, ZMS, MHS, XYZ, XC, XW, QW. All authors read and approved the final manuscript.

## Pre-publication history

The pre-publication history for this paper can be accessed here:

http://www.biomedcentral.com/1471-2407/13/19/prepub

## Supplementary Material

Additional file 1: Table S1 Distributions of selected variables in cervical cancer cases and cancer-free female controls. **Table S2.** Interactions between genotypes of the *LAMB3-miR-218* pathway and environmental factors on cervical cancer risk. **Table S3.** False-positive report probability values for associations between genotypes of the *LAMB3-miR-218* pathway and cervical cancer risk. Click here for file
