# Supplementary material for: A pri-miR-218 variant and risk of cervical carcinoma in Chinese women
Source: BMC Cancer. 2013 Jan 15;13:19. doi: 10.1186/1471-2407-13-19 (PMC3585813; doi:10.1186/1471-2407-13-19)
Supplement: Additional file 1: Table S1 — Distributions of selected variables in cervical cancer cases and cancer-free female controls. Table S2. Interactions between genotypes of the LAMB3-miR-218 pathway and environmental factors on cervical cancer risk. Table S3. False-positive report probability values for associations between genotypes of the LAMB3-miR-218 pathway and cervical cancer risk. [file 1471-2407-13-19-S1.doc]

| **Table S1. Distributions of selected variables in cervical cancer cases and cancer-free female controls** | | | |
| --- | --- | --- | --- |
| **Variables** | **Cases N (%)** | **Controls N (%)** | **P*** |
| All patients | 1565 (100) | 1391 (100) |  |
| Age, years (Mean±SD) | 45.8 ± 9.8 | 46.1 ± 8.9 | 0.226 |
| ≤ 46 (Mean) | 883 (56.4) | 754 (54.2) |  |
| > 46 (Mean) | 682 (43.6) | 637 (45.8) |  |
| Age at primiparity, years (Mean±SD) | 23.6 ± 3.0 | 24.4 ± 2.4 | <.0001 |
| ≤ 24 (Mean) | 933 (63.2) | 699 (51.0) |  |
| > 24 (Mean) | 544 (36.8) | 672 (49.0) |  |
| Missing | 88 | 20 |  |
| Menopausal status |  |  | <.0001 |
| Premenopausal | 1126 (72.5) | 840 (60.5) |  |
| Postmenopausal | 427 (27.5) | 549 (39.5) |  |
| Missing | 12 | 2 |  |
| BMI, kg/m^2^ |  |  | <.0001 |
| < 25 | 1201 (78.2) | 916 (65.9) |  |
| ≥ 25 | 335 (21.8) | 474 (34.1) |  |
| Missing | 29 | 1 |  |
| Histology |  |  |  |
| CINIII | 161 (10.3) |  |  |
| SCC | 1238 (79.4) |  |  |
| Adenocarcinoma | 103 (6.6) |  |  |
| Adenosquamous | 37 (2.4) |  |  |
| Others | 21 (1.4) |  |  |
| Missing | 5 |  |  |
| FIGO stage |  |  |  |
| I | 730 (55.4) |  |  |
| II | 539 (40.9) |  |  |
| III | 44 (3.3) |  |  |
| IV | 4 (0.3) |  |  |
| Missing | 248 |  |  |
| Tumor size, cm |  |  |  |
| < 4 | 940 (65.4) |  |  |
| ≥ 4 | 497 (34.6) |  |  |
| Missing | 128 |  |  |
| Pelvic LN |  |  |  |
| Negative | 1139 (76.6) |  |  |
| Positive | 348 (23.4) |  |  |
| Missing | 78 |  |  |
| LVSI |  |  |  |
| Negative | 882 (66.4) |  |  |
| Positive | 446 (33.6) |  |  |
| Missing | 237 |  |  |
| Depth of cervical stromal invasion | |  |  |
| ≤ 1/2 | 683 (46.7) |  |  |
| > 1/2 | 781 (53.4) |  |  |
| Missing | 101 |  |  |
| ER expression |  |  |  |
| Negative | 749 (92.2) |  |  |
| Positive | 63 (7.8) |  |  |
| Missing | 753 |  |  |
| PR expression |  |  |  |
| Negative | 787 (96.9) |  |  |
| Positive | 25 (3.1) |  |  |
| Missing | 753 |  |  |
| BMI, body mass index; CIN, cervical intraepithelial neoplasia; SCC, squamous cell carcinoma; FIGO, International Federation of Gynecology and Obstetrics; LN, lymph node; LVSI, lympho-vascular space invasion; ER, estrogen receptor; PR, progesterone receptor.  * Two-sided *χ^2^*test for distributions between cases and controls. | | | |

| **Table S2. Interactions between genotypes of the *LAMB3-miR-218* pathway and environmental factors on cervical cancer risk** | | |
| --- | --- | --- |
| **Interaction between two factors** | **OR (95% CI)** | ***P**** |
| *pri-miR-218* rs11134527 * age at primiparity | 1.17 (0.78-1.77) | 0.452 |
| *pri-miR-218* rs11134527 * menopausal status | 1.19 (0.78-1.83) | 0.425 |
| *pri-miR-218* rs11134527 * BMI | 0.92 (0.58-1.45) | 0.715 |
| *LAMB3* rs2566 * age at primiparity | 0.84 (0.52-1.34) | 0.460 |
| *LAMB3* rs2566 * menopausal status | 0.89 (0.54-1.45) | 0.635 |
| *LAMB3* rs2566 * BMI | 1.09 (0.65-1.82) | 0.739 |
| *pri-miR-218* rs11134527 * *LAMB3* rs2566 | 1.18 (0.63-2.22) | 0.603 |
| OR, odds ratio; CI, confidence interval; BMI, body mass index.  * Logistic regression analysis. | | |

| **Table S3.** **False-positive report probability values for associations between genotypes of the *LAMB3-miR-218* pathway and cervical cancer risk** | | | | | | | | |
| --- | --- | --- | --- | --- | --- | --- | --- | --- |
| **Genotypes** | **Positive OR (95% CI)*** | ***P**** | **Statistical power**** | **Prior probability** | | | | |
|  |  |  |  | **0.25** | **0.1** | **0.01** | **0.001** | **0.0001** |
| ***miR-218* rs11134527** |  |  |  |  |  |  |  |  |
| GG *vs.* AA/AG |  |  |  |  |  |  |  |  |
| All patients | 0.80 (0.66-0.98) | 0.028 | 0.949 | **0.081** | 0.210 | 0.745 | 0.967 | 0.997 |
| Age at primiparity ≤ 24 years | 0.74 (0.57-0.96) | 0.025 | 0.756 | **0.090** | 0.229 | 0.766 | 0.971 | 0.997 |
| Premenopausal | 0.75 (0.59-0.96) | 0.021 | 0.813 | **0.072** | **0.189** | 0.719 | 0.963 | 0.996 |
| SCC | 0.76 (0.61-0.94) | 0.012 | 0.864 | **0.040** | **0.111** | 0.579 | 0.933 | 0.993 |
| FIGO stage I | 0.73 (0.57-0.94) | 0.016 | 0.740 | **0.061** | **0.163** | 0.682 | 0.956 | 0.995 |
| Positive pelvic LN | 0.60 (0.42-0.86) | 0.006 | 0.299 | **0.057** | **0.153** | 0.665 | 0.953 | 0.995 |
| Positive LVSI | 0.69 (0.50-0.94) | 0.018 | 0.560 | **0.088** | 0.224 | 0.761 | 0.970 | 0.997 |
| ER-negative expression | 0.75 (0.59-0.97) | 0.026 | 0.806 | **0.088** | 0.225 | 0.762 | 0.970 | 0.997 |
| PR-negative expression | 0.78 (0.61-0.99) | 0.042 | 0.868 | **0.127** | 0.303 | 0.827 | 0.980 | 0.998 |
| OR, odds ratio; CI, confidence interval; SCC, squamous cell carcinoma; FIGO, International Federation of Gynecology and Obstetrics; LN, Lymph Node; LVSI, lympho-vascular space invasion; ER, estrogen receptor; PR, progesterone receptor.  * Crude OR and *P* value;  ** Statistical power was calculated using the number of observations in the subgroup and the OR and *P* values in this table.  The results in false-positive report probability analysis were in **bold**, if the prior probability < 0.20. | | | | | | | | |
